# Supplementary material for: DNA metabarcoding-based diet survey for the Eurasian otter (Lutra lutra): Development of a Eurasian otter-specific blocking oligonucleotide for 12S rRNA gene sequencing for vertebrates
Source: PLoS One. 2019 Dec 12;14(12):e0226253. doi: 10.1371/journal.pone.0226253 (PMC6907848; doi:10.1371/journal.pone.0226253)
Supplement: Attachment — Submitted filename: Response.docx [file pone.0226253.s015.docx]

**TO:**	Prof. Zuogang Peng, Academic Editor of *Plos One*

**FROM:**	Priyanka Kumari, Ke Dong, Kyung Yeon Eo, Woo-Shin Lee, Junpei Kimura, and Naomichi Yamamoto, authors of PONE-D-19-19235R1 “DNA metabarcoding-based diet survey for the Eurasian otter (*Lutra lutra*): Development of a Eurasian otter-specific blocking oligonucleotide for 12S rRNA gene sequencing for vertebrates”

**RE:**	Response to reviewer comments

**DATE:**	November 16, 2019

The authors thank additional comments provided from the Reviewers #1 and #2. Below, please find our responses to the reviewers’ comments. In addition to responding to the reviewers’ comments, we made a final editing of our manuscript, especially for the abstraction and introduction sections and the last paragraph of discussion section, to improve readability. The authors believe that there is no significant content change incurred due to editing. The page numbers in our responses refer to the clean version of our revised manuscript.

**Reviewer #1:**

**1. The authors have addressed all of my major concerns with the original manuscript. I think the shift to focus on the development and testing of the blocking primers has given the manuscript a clearer purpose and strengthened the results. Additionally, the authors have more adequately addressed the limitations raised during the previous review. I have minor grammatical and clarification suggestions outlined below.**

Response: The authors thank positive appraisal from the Reviewer #1.

**2. Throughout manuscript: 12S and 16S sequences were referred to as "rDNA", this should be "rRNA".**

Response: The authors believe that the term “rDNA” can work as it represents DNA (gene) that encodes rRNA (transcripts), i.e., rRNA gene. Meanwhile, the term “rRNA” without the word “gene” does not work since we sequenced DNA, not RNA. So, the authors believe that both of the terms “rDNA” and “rRNA gene” can work while the term “rRNA” without the word “gene” does not work. As the Reviewer #1 suggested, however, the term “rDNA” has been replaced with the term “rRNA gene” throughout the manuscript since it seems that the term “rRNA gene” is more commonly used than the term “rDNA” in literature. Accordingly, the title of the paper has been also changed as follows:

“DNA metabarcoding-based diet survey for the Eurasian otter (*Lutra lutra*): Development of a Eurasian otter-specific blocking oligonucleotide for 12S rRNA gene sequencing for vertebrates”

**3. Line 89: remove "respectively"**

Response: Corrected.

**4. Lines 304-307: The first paragraph of the discussion would be stronger if it focused on the main takeaway from this study (that the blocking primers are very effective).**

Response: To emphasize our development of the blocking oligonucleotide OBS1, a sentence has been added at the beginning of the first paragraph of the discussion section as follows:

“In this study, we designed a Eurasian otter-specific blocking oligonucleotide (OBS1) for their diet analyses based on 12S rRNA gene sequencing for vertebrates.” Page 16 Lines 320-321

In addition, the following sentence has been included in the conclusion section.

“In particular, we developed a Eurasian otter-specific blocking oligonucleotide (OBS1) for 12S rRNA gene sequencing for vertebrates.” Page 21 Lines 424-426

**5. Line 318: "fishes (95%) was" should be "fishes (95%) were"**

Response: Corrected.

**6. Line 321: "teleost" should be "teleosts"**

Response: Corrected.

**Reviewer #2:**

**1. The authors have taken into account the main remarks of the two reviewers, especially by changing the aim of their ms, but also by adding new tests and table (especially the very informative table 3) on the performance of the blocking oligonucleotide and its potential risk of inhibiting non target DNA amplification. Overall, the ms has been significantly improve, and is for me close to a publishable state.**

Response: The authors thank positive appraisal from the Reviewer #2.

**2. But I have a last concern on the statistical tests used for testing library and loci effect. Maybe I did not understand fully how the authors used the permanova, but authors should give more information of test outputs, and maybe rethink these tests and/or how they interpret it. Alternatively, I have made proposals to test (in my opinion) the words of authors:**

Response: The authors agree that the test results of PERMANOVA cannot tell what we insisted regarding the levels of intra- vs. inter-group variabilities. The additional statistical analyses were performed as the Reviewer #2 suggested. For details, please see our response below.

**3. To demonstrate that “variabilities in the memberships of the detected fish genera were smaller across the sequencing loci (i.e., 12S rDNA vs. COI) than across the libraries (p<0.05)”**

**I would compare the mean intra-sample distances (between the two loci) vs mean intra-library distances. But I think (see my remark below) that it will be better to compare in fact these three distances: Intra-samples distances (between loci) vs inter-samples distances using COI vs inter-samples distances using 12S**

Response: The suggested analysis has been performed. Specifically, we made a boxplot showing the distributions of the pair-wise intra-sample distances obtained by 12S rRNA vs. COI gene sequencing, the inter-sample distances obtained by 12S rRNA gene sequencing, and the inter-sample distances obtained by COI gene sequencing (Fig. 8b). The intra-sample distances were smallest among other distances. In particular, they were significantly smaller than the inter-sample distances based on 12S rRNA gene sequencing. We believe that these results support our claim though they were not significantly smaller than the inter-sample distances based on COI gene sequencing. To describe these statistical results, the following sentences have been added:

“Fig. 8 shows that the variabilities in the memberships of detected fish genera were smaller across the sequencing loci (i.e., 12S rRNA gene vs. COI) than across the samples, with a statistical significance observed between the intra-sample distances based on the two different loci and the inter-sample distances based on 12S rRNA gene sequencing, indicating that the detected fish genera are in agreement between the two locus analyses in terms of detected fish memberships.” Page 16 Lines 301-306

**4. To demonstrate that “we confirmed that the variabilities in unique sequence structures were smaller across the library preparation methods (i.e., with vs. without OBS1) than across the samples”**

**I would compare the mean between-sample distances vs mean intra-library distances.**

Response: The suggested analysis has been performed by making a boxplot showing the distributions of the pair-wise intra-sample distances obtained with vs. without blocking oligonucleotides OBS1, the inter-sample distances obtained with OBS1, and the inter-sample distances obtained without OBS1 (Fig. 3b). The beta dispersion was smallest for the intra-sample group. We believe that these results support our claim.

**5. Remark:**

**L. 168 You define here what you call “unique sequence”. As you will usually use this term in the rest of the ms (result part mostly), and because the sequence-centered view is not “familiar” for metabarcoding process, I propose you to quickly remember what you stipulate by this term when you use it.**

Response: The following explanation was added for the term “unique sequences”.

“First, identical reads were binned to generate a set of unique sequences, each with an identical length and nucleotide sequence.” Page 9 Lines 167-168

**6. L.185 Maybe PCoA should be announced at this step.**

Response: The information was included in the section of statistical analyses. Additionally, we included the information of methods to analyze beta dispersion. The section has been revised as follows:

“R version 3.6.0 was used for statistical analyses. The vegan package version 2.5-6 [28] was used to check for possible changes in the sequence structures by the blocking oligonucleotides OBS1 and for the difference in fish memberships detected by sequencing of two different loci of 12S rRNA and COI genes. For the first purpose, the Bray-Curtis dissimilarities of unique sequence structures were characterized and compared across the 12S rRNA gene libraries prepared with vs. without blocking oligonucleotides OBS1 and HomoB after excluding the sequence reads assigned to Lutra lutra and Homo sapiens. For the second purpose, the Jaccard distances were calculated to characterize the differences of memberships of fish genera detected by 12S rRNA vs. COI gene sequencing analyses. Principal coordinate analysis plot and cluster dendrogram were created based on the Bray-Curtis and Jaccard distances calculated for the first and second purposes, respectively. Permutational multivariate analysis of variance (PERMANOVA) tests were conducted and followed by Kruskal-Wallis rank sum tests with post hoc Wilcoxon rank-sum tests with Bonferroni correction to compare pair-wise intra- and inter-sample distances to assess the degrees of variabilities across the samples and across the library preparation methods. P-values less than 0.05 were considered statistically significant.” Page 10 Lines 174-188

**7. L.312-316 I am confused with your conclusions. If you tested both gene and library effect on jaccard dissimiliraty matrix, and that the result is significant, it does not obligatory indicated that gene effect is larger than library effect. For that, you may indicated their relative explained variance using the R2 if you use Adonis function of vegan package. But the output of permanova is lacking in your ms, and we don’t know to what corresponded your significant value.**

Response: The authors thanks the Reviewer #1 to remind us the importance of showing the effect sizes, which are perhaps more important than *p*-values. Now, the effect sizes in terms of r^2^ were included in the revised Figs. 3 and 8.

**8. Also, maybe using betadisper function that estimate the mean distance of individuals around barycenter should be serve to propose a proxy that indicate the amount of dispersion for a grouping factor. In this case, you may calculate the global dispersion among individuals for 12S, and the global dispersion for COI. These two values could be indicate in parallel to your permanova test. To illustrate this dispersion, maybe you can boxplot the jaccard pairwise-distance among specimens for each loci, I think that it should more support your words than the dendogram.**

Response: Please see our response to the Reviewer #2’s 3rd comment above.

**9. But I have a more profound remark on your reasoning. If you prove that library dispersion is higher than loci dispersion, it does not prove that result obtained using different loci are congruent. In my sense, you have to adding the specimen effect. Loci effect may be overlooked if the mean intra-sample (between loci result of a same specimen) distance is lower than the inter-sample distance obtained with each loci.**

Response: As suggested, we compared the differences in distances in three different groups, i.e., intra-sample (12S vs. COI), inter-sample (12S), and inter-sample (COI). For details, please see our response to the Reviewer #2’s 3rd comment above.

**10. L.338-341 As it is a similar analyses, see my remark L.312-316**

Response: In Fig. 3, *r*^2^ was included.
